# Integrative Proteomic and Metabolomic Analyses Reveal Arginase-1-Mediated Nitrogen Metabolic Reprogramming Associated with Growth Advantage in Ronglai Binary Hybrid Pigs

**DOI:** 10.3390/ani16152344

**Published:** 2026-08-01

**Authors:** Wei Yang, Lan Wu, Hongyu Xie, Ding Zhang, Yuqin Wang, Jiajia Pan, Xinyi Xu, Xiaoqing Bai, Wei Zhang, Yueting Zheng

**Affiliations:** 1 Chongqing Academy of Animal Sciences, Rongchang, Chongqing 402460, China; yangw@cqaa.cn; 2 Chongqing Seed Pig Farm Co., Ltd., Rongchang, Chongqing 402460, China; 3 Key Laboratory of Animal Biochemistry and Nutrition, Ministry of Agriculture, Key Laboratory of Veterinary Biotechnology of Henan Province, College of Veterinary Medicine, Henan Agricultural University, Zhengzhou 450046, China; wl999633@stu.henau.edu.cn (L.W.); xiehongyu@stu.henau.edu.cn (H.X.); dingzhang@stu.henau.edu.cn (D.Z.); wangyuqin@stu.henau.edu.cn (Y.W.); first2017@henau.edu.cn (J.P.); zhangweiest@126.com (W.Z.); 4 Center for Biomedicalphotonics and Molecular Imaging, Xi’an Key Laboratory of Intelligent Sensing and Regulation of Trans-Scale Life Information, School of Life Science and Technology, Xidian University, Xi’an 710126, China; xyxu@xidian.edu.cn

**Keywords:** Rongchang pigs, Ronglai binary hybrid pigs, arginase-1, nitrogen metabolic reprogramming, growth advantage

## Abstract

Chinese native pig breeds have outstanding advantages in meat quality and adaptability but often exhibit slower growth and lower feed efficiency, limiting their commercial utilization. How to improve the growth efficiency of local pigs while maintaining their excellent characteristics through hybridization and mechanism analysis is the key to improving the quality and efficiency of the current industry. This study systematically evaluated the growth advantage of Ronglai binary hybrid pigs (RS, derived from Rongchang × Laiwu crosses) and integrated proteomic and metabolomic analyses to uncover the underlying metabolic mechanisms. Compared with purebred Rongchang pigs (RR), RS pigs showed a significantly higher average daily gain (553 vs. 454 g/d) from 85 days of age to the 90 kg body weight stage, representing a 21.8% improvement. Integrated multi-omics analysis revealed marked downregulation of arginase-1 (ARG1) protein in RS pigs, accompanied by decreased ornithine levels and elevated amounts of arginine-containing dipeptides. The elevated plasma NOx levels observed in RS pigs suggest that arginine accumulation may activate the NOS pathway. This reprogramming of nitrogen metabolism is associated with improved dietary nitrogen retention and utilization, thereby potentially directing more nutrients toward growth. While our findings provide a link between the growth advantage of this local pig cross and ARG1-mediated nitrogen metabolic remodeling, we acknowledge that to formally distinguish between heterosis and additive genetic effects, a control group of pure Laiwu pigs needs to be included.

## Simple Summary

Chinese local pig breeds have excellent meat quality but often grow slowly. We studied Rong-Lai hybrid pigs, which combine Rongchang and Laiwu breeds, to understand why they grow faster than purebred pigs. These hybrid pigs gained weight 20% faster than purebred Rongchang pigs. With integrated proteomic and metabolomic analyses, we found that ARG1 downregulation is associated with arginine accumulation and activation of the NOS pathway, as evidenced by increased plasma NOx. Our findings show how hybridization-associated metabolic changes may contribute to improved growth, providing insights for breeding programs aimed at developing more efficient pigs while maintaining good meat quality. However, we acknowledge that the absence of a pure Laiwu control group precludes definitive attribution of these effects to heterosis versus additive genetic contributions.

## 1. Introduction

Chinese local pig breeds, such as Rongchang pigs, Laiwu pigs and Taihu pigs, are valuable genetic resources with unique advantages, including excellent meat quality, strong stress resistance and high adaptability [1,2,3]. However, with the widespread promotion of commercial pig breeds (e.g., Landrace and Duroc pigs), which were strongly selected for skeletal muscle-related traits like daily gain, local pig breeds face challenges of slower growth and lower feed conversion ratio, leading to population decline and loss of genetic diversity.

The Rongchang pig is one of the excellent local pig breeds in China, mainly distributed in southwest areas such as Sichuan and Chongqing, with the advantages of delicious meat and strong adaptability. Laiwu pigs are mainly distributed in Laiwu, Shandong Province, and are famous for their roughage tolerance, strong disease resistance and excellent meat quality [4], but their growth rate is relatively slow. Although Laiwu pigs exhibit inferior growth performance compared with certain improved pig breeds, their exceptional disease resistance and adaptability render them an ideal dam for hybridization [5,6].

Hybridization is a widely used method for improving livestock production performance by combining the desirable traits of both parents, leading to heterosis of their offspring [7,8]. Heterosis typically manifests as faster growth rates, enhanced disease resistance and improved feed conversion efficiency [9]. For example, a hybridization study in Laiwu pigs and Tibetan pigs revealed upregulated expression of immune-related genes (e.g., TLR4 and IFN-γ) in hybrid offspring, along with significantly higher serum IgG levels compared with parental lines. These findings demonstrate that hybridization enhances innate immune responses. In summary, hybridization technology is widely implemented in swine production, substantially improving both production efficiency and economic returns [9,10]. Hybridization can also reduce the excessive selection pressure on purebred local pigs, aiding in population conservation and sustainable utilization [11]. As two elite indigenous pig breeds, Rongchang and Laiwu pigs exhibit complementary genetic strengths. Rongchang pigs demonstrate superior growth rates, whereas Laiwu pigs are characterized by robust disease resistance and environmental adaptability. Strategic hybridization of Rongchang sires with Laiwu dams generates RongLai hybrid pigs, which synergistically combine these parental traits, resulting in significant heterosis manifested through enhanced growth performance, improved disease resilience, and strengthened adaptive capacity.

Growth performance is one of the key economic traits in pig farming, affecting the economic benefits of farmers directly [12,13]. In recent years, with the development of molecular biology, researchers have begun to explore the mechanisms influencing pig growth performance at the molecular level. Proteome analysis, as a high throughput sequencing technology, enables comprehensive analysis of protein expression and function in organisms, providing a powerful tool for uncovering the molecular mechanisms of growth regulation [14,15]. Through proteome analysis, differentially expressed proteins (DEPs) related to growth traits can be identified, and their functions in signaling pathways can be further analyzed. For example, the Wnt signaling pathway [16], the PI3K-Akt signaling pathway [17] and the mTOR signaling pathway [18,19,20] play crucial roles in cell proliferation, metabolic regulation, and growth and development. By studying changes in protein expression within these pathways, the molecular basis of heterosis in hybrid pigs can be better understood. In summary, our study breeds Ronglai binary hybrid pigs by crossing Rongchang pigs with Laiwu pigs, aiming to explore the growth performance advantage and its underlying molecular mechanisms. This research study not only provides a theoretical basis for the breed improvement of Rongchang pigs but also helps enhance their market competitiveness, promoting the sustainable development of pig farming.

## 2. Materials and Methods

### 2.1. Experimental Animals and Grouping

Ronglai binary hybrid pigs (RS) were generated by crossing Rongchang sows (maternal line) with semen from Laiwu boars (paternal line). All animals were obtained from the National Core Breeding Farm of Chongqing Qitai Jiamu Breeding Co., Ltd. (Chongqing, China), and were reared under standardized conditions with identical feeding regimes, nutritional levels, and management practices throughout the experiment.

A total of 200 healthy male castrated pigs were used in this study, with 100 individuals assigned to each of the two experimental groups: purebred Rongchang pigs (RR group) and Ronglai binary hybrid pigs (RS group). Inclusion criteria were: (1) clinically healthy status confirmed by veterinary examination and (2) no history of illness or medical treatment during the experimental period. Exclusion criteria were: (1) any signs of illness or abnormal behavior and (2) incomplete growth records. Animals were randomly selected from a larger cohort for multi-omics analysis using a computer-generated random number sequence (Excel RAND function). For plasma sample processing, samples were randomized and processed in a randomized order to minimize batch effects. Laboratory personnel performing proteomic and metabolomic analyses were blinded to group allocation; samples were coded and group identities were revealed only after data acquisition and initial quality control. Body weights were recorded at two key time points: at the end of 85 days of conservation and when 90 kg body weight was reached.

For multi-omics analysis, blood samples were collected from 6 randomly selected pigs per group at 90 kg body weight of age via anterior vena cava puncture using EDTA-coated tubes. Sample size was determined based on the following considerations: (1) this sample size is consistent with published deep plasma proteomic studies using Orbitrap Astral™ platforms (Thermo Fisher Scientific, Waltham, MA, USA), where typical group sizes range from 5 to 8 biological replicates; (2) resource constraints associated with ultradeep proteomic profiling limited feasible sample throughput while maintaining comprehensive coverage. Approximately 3 mL of whole blood was collected from each animal, gently inverted immediately after collection, and centrifuged at 3000 rpm for 10 min at 4 °C. The supernatant (plasma) was aliquoted (200 μL per tube), flash frozen in liquid nitrogen, and stored at −80 °C until subsequent proteomic and metabolomic analyses.

All animal procedures were conducted in strict accordance with the guidelines and regulations of the Animal Welfare and Ethics Committee of Chongqing Academy of Animal Sciences and were approved by the Institutional Animal Care and Use Committee (IACUC) of Chongqing Academy of Animal Sciences (Approval No. CQAAS-IACUC-2025-015, approved on 4 March 2025).

### 2.2. Growth Performance Analysis

Average daily gain (ADG) was used to analyze growth traits. ADG was calculated using the formula: ADG = (final weight − initial weight)/number of feeding days.

Statistical software (SPSS 26.0) was used to perform *t*-tests on the body weight data of the two groups to analyze the significance of differences. The growth advantage rate for average daily gain was calculated using the standard formula: The growth advantage rate (%) = [(RS mean − RR mean)/RR mean] × 100, where RS represents the F1 crossbred population (Rongchang × Laiwu) and RR represents the purebred Rongchang population. This calculation provides an estimate of the relative improvement in growth performance of the crossbred offspring compared with the maternal purebred line. We acknowledge that a complete heterosis calculation would ideally include both parental lines (RR and LL), as discussed in Section 4.

### 2.3. Proteome Analysis

All samples were sent to Wuhan Metware Biotechnology Co., Ltd. (Wuhan, China), for ultradeep blood proteome analysis using functional biomagnetic bead-based enrichment of low-abundance proteins and Orbitrap™ Astral™ mass spectrometry DIA scanning. Briefly, plasma proteomic profiling was performed using an ultradeep LC-MS/MS platform. Proteins were extracted from plasma, and low-abundance proteins were enriched using the EasyPept™ DeeP kit (Shanghai e-Calculation Biotechnology Co., Ltd., Shanghai, China) with functionalized nanomagnetic beads. After tryptic digestion, peptides were separated on a Vanquish Neo UHPLC system coupled online to an Orbitrap Astral™ mass spectrometer (Thermo Fisher Scientific, Waltham, MA, USA). Data-independent acquisition (DIA) was employed with MS1 resolution set to 240,000 and 299 DIA windows.

Raw data were processed using DIA-NN (v1.8.1) in library-free mode against the Sus scrofa UniProtKB database. Match-between-runs (MBR) was enabled, and false discovery rate (FDR) was controlled at ≤1%. Label-free quantification was performed using the MaxLFQ algorithm. Proteins with |fold change| ≥ 1.5 and *p* < 0.05 (Student’s *t*-test) were defined as differentially expressed. Functional enrichment analysis of Gene Ontology (GO) terms and KEGG pathways was conducted using clusterProfiler (v4.4.4) with a significance threshold of *p* < 0.05.

Proteins with |fold change| ≥ 1.5 and q < 0.10 (Benjamini–Hochberg FDR-adjusted *p*-value) were defined as differentially expressed proteins (DEPs). This FDR threshold balances the need to control false positives in the context of >6000 quantified proteins while maintaining sensitivity to detect biologically meaningful changes in this exploratory multi-omics study.

The mass spectrometer was calibrated daily using standard calibration solutions according to the manufacturer’s protocols, and instrument performance was verified by analyzing a standard peptide mixture before each batch. Only runs meeting predefined acceptance criteria (mass accuracy < 3 ppm, retention time drift < 0.5 min, and coefficient of variation for QC samples < 15%) were included in the final analysis.

### 2.4. Metabolomic Analysis

Plasma metabolomic profiling was performed using a widely targeted metabolomics approach on an ultraperformance liquid chromatography–tandem mass spectrometry (UPLC-MS/MS) platform. Briefly, metabolites were extracted from plasma using a 20% acetonitrile–methanol solution containing internal standards. Chromatographic separation was achieved on a T3 C18 column or a HILIC column in positive or negative ionization mode, respectively, using mobile phases containing 0.1% formic acid or 20 mM ammonium formate (pH 10.6). Mass spectrometry analysis was conducted on a QTRAP^®^ 6500+ system (SCIEX) in multiple reaction monitoring (MRM) mode for precise quantification.

Raw data were processed using MultiQuant 2.0 software. Metabolites were identified by matching retention times and MS/MS spectra against the self-constructed MWDB database and public databases (KEGG and HMDB). Quality control was ensured by calculating coefficient of variation (CV) for QC samples, and only metabolites with CV < 0.3 were retained. Differential metabolites between groups were identified based on variable importance in projection (VIP) > 1 from orthogonal partial least squares discriminant analysis (OPLS-DA) and Student’s *t*-test *p*-value < 0.05. Functional annotation and pathway enrichment analysis were performed using the KEGG database. The extraction, detection, and quantitative analysis of metabolite profiling were performed by Wuhan Metware Biotechnology Co., Ltd. (www.metware.cn, accessed on 20 July 2025.).

Differential metabolites between groups were identified based on variable importance in projection (VIP) > 1 from orthogonal partial least squares discriminant analysis (OPLS-DA) and Student’s *t*-test *p*-value < 0.05. Additionally, the Benjamini–Hochberg false discovery rate (FDR) procedure was applied to correct for multiple comparisons across all tested metabolites, and metabolites with q < 0.10 were considered statistically significant. Instrument calibration was performed using standard solutions before each batch, and mass accuracy was verified using reference standards. Metabolites with a coefficient of variation (CV) > 0.3 in QC samples were excluded from further analysis.

### 2.5. Measurement of Plasma Total Nitrite and Nitrate (NOx) by Griess Assay

To evaluate nitric oxide (NO) production in vivo, total nitrite and nitrate (NOx) levels were determined in plasma samples using a Griess reagent kit (Beyotime Biotechnology, Shanghai, China; Cat# S0023). Plasma aliquots (100 μL) stored at −80 °C were thawed on ice and centrifuged at 12,000× *g* for 10 min at 4 °C to remove precipitated proteins. The supernatant was filtered through a 10 kDa molecular weight cut-off filter (Millipore, Burlington, MA, USA) to eliminate protein interference. Because the Griess reaction directly detects nitrite (NO_2_^−^), nitrate (NO_3_^−^) was first reduced to nitrite by adding nitrate reductase (0.2 U/μL) and NADPH (50 μM) to 50 μL of filtered plasma. The mixture was incubated at 37 °C for 30 min, followed by addition of 10 μL of lactate dehydrogenase (5 U/mL) and pyruvate (10 mM) to consume the remaining NADPH, thereby avoiding interference with the subsequent colorimetric reaction.

After reduction, 50 μL of the processed sample was mixed with 50 μL of Griess Reagent I (0.1% N-1-naphthylethylenediamine dihydrochloride) and 50 μL of Griess Reagent II (1% sulfanilamide in 5% phosphoric acid) in a 96-well plate. The reaction was incubated at room temperature for 10 min in the dark, and absorbance was measured at 540 nm using a microplate reader (Bio-Tek, Shoreline, WA, USA). A standard curve was prepared using sodium nitrite (NaNO_2_) at concentrations ranging from 0 to 100 μM. The assay was validated for use with pig plasma. The limit of detection (LOD) was determined as the mean blank signal plus 3.3 times the standard deviation of the blank (LOD = 0.15 μM), and the limit of quantification (LOQ) was defined as the mean blank signal plus 10 times the standard deviation of the blank (LOQ = 0.50 μM). The assay showed linearity across the range of 0.5–100 μM (R^2^ > 0.998). All plasma NOx measurements fell within the linear range of the standard curve. Spike-recovery experiments (addition of 10 μM and 50 μM NaNO_2_ to plasma samples) yielded recoveries of 94.3 ± 3.6% and 96.8 ± 2.9%, respectively, confirming assay accuracy. The intra-assay coefficient of variation (CV) was <5%, and the inter-assay CV was <8%. Total NOx concentration in each plasma sample was calculated by interpolation from the standard curve and expressed as micromolar (μM). All samples were assayed in duplicate, and the intra-assay coefficient of variation (CV) was below 5%.

### 2.6. Statistical Analysis

All statistical analyses were performed using SPSS (IBM Corp., Armonk, NY, USA). Before applying parametric tests, the assumptions of normality and homogeneity of variances were assessed. Normality was evaluated using the Shapiro–Wilk test (*p* > 0.05 considered normal distribution) and Q–Q plots. Homogeneity of variances between groups was assessed using Levene’s test (*p* > 0.05 indicated equal variances). For comparisons between two groups, when data met both assumptions, an unpaired two-tailed Student’s *t*-test was applied. When the normality assumption was violated, the non-parametric Mann–Whitney U test was used as an alternative. For proteomic and metabolomic data, Welch’s *t*-test (which does not assume equal variances) was applied when Levene’s test indicated unequal variances. All data are presented as means ± SEM unless otherwise specified.

## 3. Results

### 3.1. Growth Performance of Ronglai Binary Hybrid Pig

**Figure 1:**
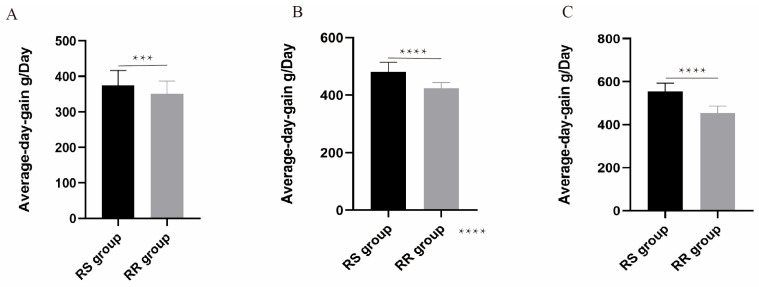
Comparison of growth performance between Rongchang pigs and Ronglai binary hybrid pigs. (**A**–**C**) Bar graphs show the average daily gain (ADG, g/d) of the Rongchang (RR) and Ronglai hybrid (RS) groups during the 0–85-day (**A**) and 0–90 kg periods (**B**) and from the 85th day to 90 kg (**C**). Individual data points are shown to illustrate the distribution within each group. Error bars represent means ± SEM. **** *p* < 0.0001; *** *p* < 0.001.

To evaluate the growth performance, this study systematically measured the body weight and analyzed average daily gain (ADG) of Ronglai binary hybrid pigs (RS) at key growth stages, using purebred Rongchang pigs (RR) as the control. Results from 100 castrated male pigs per breed showed that the RS group exhibited a significant growth advantage at two measured stages compared with the RR group. During the period from 0 to 85 days, the ADG of the RS group reached 374 g/d, significantly higher than the 350 g/d of the RR group (Figure 1A). From 0 to 90 kg, the overall ADG of the RS group was 480 g/d, a significant increase compared with 423 g/d in the RR group (Figure 1B). From the 85th day to 90 kg, the overall ADG of the RS group was 553 g/d, a significant increase compared with 454 g/d in the RR group (Figure 1C). These results indicate that compared with the paternal Rongchang pigs, the Ronglai binary hybrid pigs demonstrated a stable and significantly increased growth rate throughout the observation period, clearly reflecting the growth-enhancing effect of this specific cross. The growth advantage rate for ADG from 85 days to 90 kg was calculated as 21.8%.

### 3.2. Integrated Multi-Omics Analysis

To investigate the molecular mechanisms underlying the growth advantage of Ronglai binary hybrid pigs (RS), we performed proteomic analyses (Supplementary Figure S1) and metabolomic analyses (Supplementary Figure S2) from both RR and RS groups.

**Figure 2:**
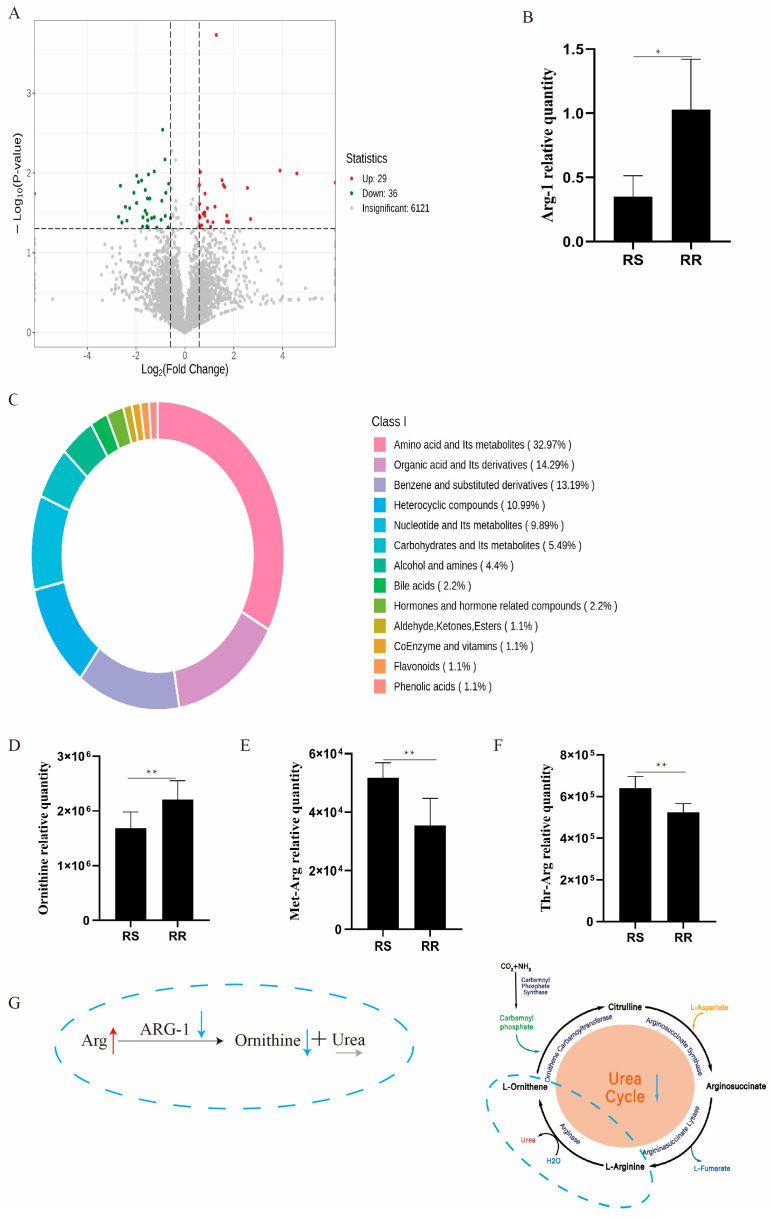
Integrated proteomic and metabolomic analyses. (**A**) Volcano plot of differentially expressed proteins (DEPs) between RR and RS groups. (**B**) Expression level of arginase-1 (ARG1). (**C**) Classification of differential metabolites. (**D**–**F**) Relative abundance of ornithine (**D**), threonine arginine dipeptide (**E**), and methionine arginine dipeptide (**F**). (**G**) Schematic summary of key changes in the urea cycle-related pathway. Error bars represent means ± SEM. * *p* < 0.05 and ** *p* < 0.01. Red and ↑ indicate substances or pathways significantly increased or activated in RS pigs; blue and ↓ indicate substances or pathways significantly decreased or inhibited in RS pigs; gray and → indicate no significant changes between RS and RR groups.

Proteomic analysis quantified 6121 proteins, and differential expression analysis (RS vs. RR) identified 65 significantly differentially expressed proteins (DEPs) (Figure 2A). Among the DEPs, 29 proteins were upregulated, and 36 were downregulated in the RS group. Notably, arginase-1 (ARG1) was significantly downregulated in the RS group (FC = 2.944, *p* = 0.0327) (Figure 2B). Concurrently, metabolomic analysis screened 114 significantly differential metabolites, of which amino acid-related metabolites accounted for 32.97% (Figure 2C). Among the altered metabolites, ornithine, closely associated with the urea cycle, was significantly reduced in the RS group (VIP = 1.84, *p* = 0.025) (Figure 2D). In contrast, two arginine-containing dipeptides, threonine arginine (VIP = 2.15, *p* = 0.005) (Figure 2E) and methionine arginine (VIP = 1.73, *p* = 0.037), were significantly accumulated in the RS group (Figure 2F). These metabolite changes align closely with the observed downregulation of ARG1 in the proteomic data, collectively indicating a weakened urea cycle in RS pigs (Figure 2G).

### 3.3. Proposed Hypothesis of Nitrogen Metabolic Reprogramming

**Figure 3:**
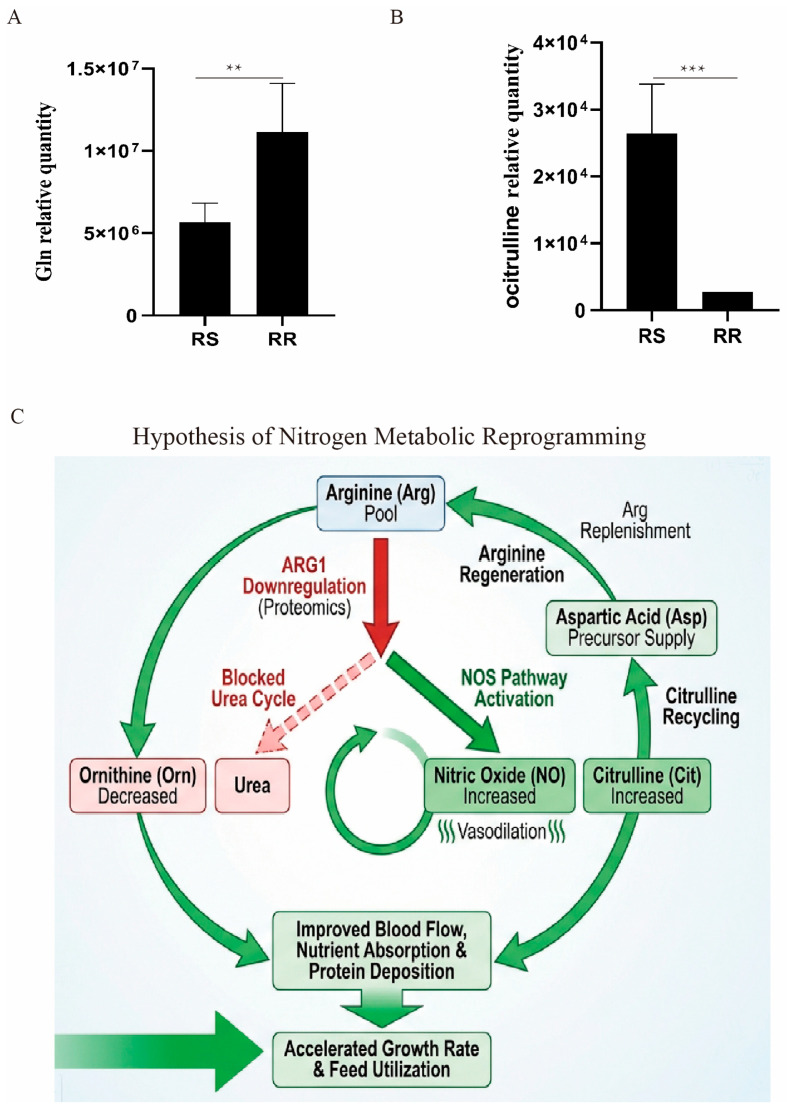
Proposed hypothesis of nitrogen metabolic reprogramming in Ronglai hybrid pigs. (**A**) Relative changes in glutamine in RS pigs compared with RR pigs. (**B**) Relative changes in ocitrulline in RS pigs compared with RR pigs. (**C**) Proposed “throttle, activate, recycle” model of hepatic nitrogen network reprogramming. Downregulation of ARG1 (“throttle”) reduces ornithine production and urea cycle flux, leading to arginine accumulation. Increased arginine activates the NOS pathway (“activate”), producing NO and citrulline. To recycle citrulline and maintain nitrogen homeostasis, glutamine reserves are mobilized to provide aspartate, driving conversion of citrulline back into arginine (“recycle”). This reallocation directs nitrogen resources toward growth-supporting pathways. Error bars represent means ± SEM. *** *p* < 0.001 and ** *p* < 0.01.

Glutamine, the most abundant free amino acid in the body, serves as the primary nitrogen carrier and reserve. Metabolomic analysis revealed a significant reduction in glutamine levels in RS pigs, which contrasted sharply with the marked increase in arginine. This shift suggests a reallocation of nitrogen resources from a “storage form” toward a more “active form” (Figure 3A).

Based on these observations, we propose a “throttle, activate, recycle” model of hepatic nitrogen metabolic reprogramming that underlies the growth advantage in Ronglai binary hybrid pigs (Figure 3B). Specifically, throttling (downregulation of the main pathway): downregulation of arginase-1 (ARG1↓) effectively closes the major “consumption valve” that directly breaks down arginine into urea; activating (upregulation of a bypass pathway): accumulated arginine activates the NOS (nitric oxide synthase) bypass, promoting the production of NO (nitric oxide, which may improve blood circulation to support growth) and citrulline (significantly increased); recycling and resource reallocation (systemic adjustment): to process the increased citrulline and maintain nitrogen homeostasis, the organism mobilizes glutamine reserves to generate aspartate, thereby driving the efficient recycling of citrulline back into arginine. This process maintains urea output while reallocating nitrogen resources from the glutamine pool to the arginine pool.

Importantly, while the integrated data are consistent with this model, we acknowledge that the proposed pathways are inferred from correlative omics data and require functional validation. The model is therefore presented as a working hypothesis rather than a proven mechanism.

### 3.4. Elevated Plasma NOx Levels in Ronglai Hybrid Pigs

**Figure 4:**
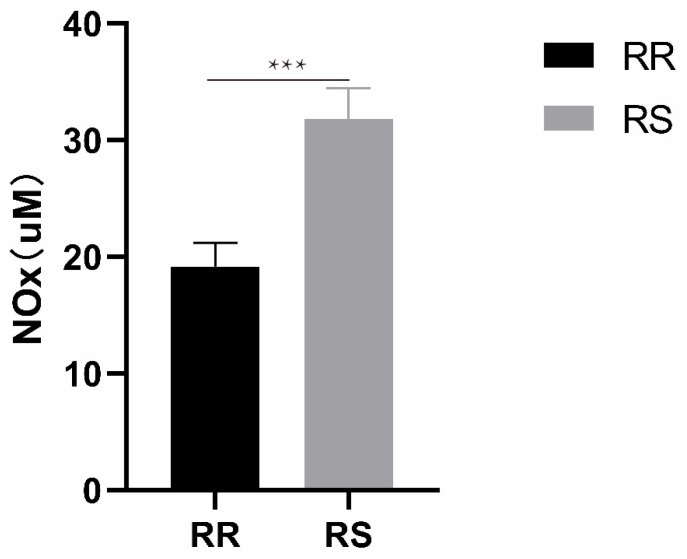
Plasma total nitrite and nitrate (NOx) levels in RR and RS pigs. Error bars represent means ± SEM. *** *p* < 0.001.

To test whether arginine accumulation activates the NOS pathway in vivo, we measured plasma total nitrite and nitrate (NOx) levels as a surrogate for NO production using the Griess assay. As shown in Figure 4, RS pigs exhibited significantly higher plasma NOx concentrations compared with RR pigs (31.8 ± 3.6 μM vs. 19.2 ± 2.4 μM). This approximately 66% increase in circulating NOx indicates enhanced systemic NOS activity in RS pigs, providing direct experimental support for the “activate” component of the proposed nitrogen metabolic reprogramming model. The assay validation parameters (LOD, LOQ, linearity, and spike recovery) are described in Section 2.5, and all measurements fell within the validated linear range.

## 4. Analysis and Discussion

In this study, we systematically evaluated the growth performance of Ronglai binary hybrid pigs (RS) derived from Rongchang sires and Laiwu dams and integrated proteomic and metabolomic analyses to uncover the molecular mechanisms underlying their growth advantage. Compared with purebred Rongchang pigs (RR), RS pigs exhibited a significantly higher average daily gain (ADG) from 85 days of age to 90 kg body weight (553 vs. 454 g/d, representing a 21.8% growth advantage rate). This consistent growth superiority provides a robust phenotypic basis for subsequent multi-omics investigation.

Our proteomic analysis identified 65 differentially expressed proteins between RS and RR pigs, among which arginase-1 (ARG1) was markedly downregulated in RS pigs. ARG1 is a key urea cycle enzyme that hydrolyzes arginine to ornithine and urea. The decreased ARG1 protein level was accompanied by significantly reduced plasma ornithine and elevated levels of arginine-containing dipeptides (threonine arginine and methionine arginine), indicating a diminished urea cycle flux. Metabolomic analysis further revealed a notable reduction in glutamine, the body’s primary nitrogen carrier and reserve, coupled with increased arginine. Collectively, these changes are consistent with reprogrammed nitrogen metabolism in RS pigs, where nitrogen resources are redirected from urea excretion toward the arginine pool.

An important nuance deserves discussion: while free arginine itself did not reach statistical significance, arginine-containing dipeptides (Thr-Arg and Met-Arg) were significantly elevated, and plasma NOx was markedly increased. We interpret this pattern as evidence of rapid metabolic channeling. Specifically, the arginine spared from urea cycle degradation appears to be rapidly diverted toward the NOS pathway, as directly demonstrated by elevated NOx. Additionally, arginine-containing dipeptides may serve as a “buffer pool” that sequesters arginine, protecting it from rapid degradation while maintaining bioavailability for downstream pathways. The accumulation of these dipeptides could also reflect changes in protein turnover or intracellular peptide metabolism. While the exact dynamics of free arginine, dipeptide pools, and NOS flux require further investigation through isotopic tracing studies, the integrated evidence collectively supports a model of nitrogen redistribution toward the NOS pathway.

To test whether the accumulated arginine activates the nitric oxide synthase (NOS) pathway—a major alternative route of arginine metabolism—we measured plasma total nitrite and nitrate (NOx) as a surrogate for NO production using the Griess assay. RS pigs showed significantly higher NOx levels than RR pigs (31.8 ± 3.6 vs. 19.2 ± 2.4 μM), providing direct evidence for NOS pathway activation in vivo. Importantly, NO is a pleiotropic signaling molecule that may promote growth through multiple well-established mechanisms: (1) NO-mediated vasodilation enhances blood flow to skeletal muscle and other metabolically active tissues, thereby improving the delivery of amino acids, glucose, and oxygen required for protein synthesis [21]; (2) NO can activate the mTORC1 pathway via cGMP/PKG-dependent or S-nitrosylation-dependent mechanisms, directly accelerating translation and muscle hypertrophy [22]; and (3) NO induces mitochondrial biogenesis via PGC-1α, improving energy efficiency [23]. These NO-driven anabolic effects provide a plausible mechanistic link between ARG1 downregulation and the superior growth rate of RS pigs.

Based on these integrated findings, we propose a “throttle, activate, recycle” model of hepatic nitrogen metabolic reprogramming that underlies the growth advantage of Ronglai binary hybrid pigs: Throttling: Downregulation of ARG1 reduces the conversion of arginine to ornithine and urea, effectively closing the major “consumption valve” of the urea cycle. Activation: The resulting arginine accumulation shifts metabolism toward the NOS pathway, producing NO and citrulline. The elevated NO is hypothesized to promote growth via vasodilation, mTOR activation, satellite cell proliferation, myostatin suppression, and mitochondrial biogenesis. Recycling: To maintain nitrogen homeostasis, glutamine reserves are mobilized to provide aspartate, driving the efficient recycling of citrulline back into arginine. This reallocation directs nitrogen resources from storage pools (glutamine) toward anabolic pathways centered on arginine and its downstream effectors.

First and foremost, the absence of purebred Laiwu (LL) pigs as a control group represents a significant limitation of our experimental design. With only two genetic groups (RR and RS), we cannot formally distinguish between true heterosis (non-additive genetic effects) and additive genetic contributions from the Laiwu breed. While our study clearly demonstrates that RS pigs exhibit a growth advantage over RR pigs and that this advantage is associated with ARG1-mediated nitrogen metabolic reprogramming, we cannot definitively attribute this phenotype to heterosis per se. A complete diallel design incorporating all three genetic groups (RR, LL, and RS) would be required to partition additive versus non-additive genetic effects and to establish whether the observed metabolic remodeling represents true heterosis or breed-specific inheritance. Therefore, our findings should be interpreted as associations with the crossbred phenotype rather than definitive evidence of heterosis mechanisms.

It is important to emphasize that our findings are primarily correlative and do not establish causality. While the integrated proteomic, metabolomic, and NOx data are consistent with the proposed model, we did not directly measure NOS enzymatic activity, mTORC1 phosphorylation status, satellite cell proliferation, myostatin levels, or rates of muscle protein synthesis. Therefore, the “throttle–activate–recycle” model should be regarded as a hypothesis generated from multi-omics observations that requires rigorous functional validation through targeted mechanistic studies. This distinction is critical: our data provide strong associative evidence and a testable mechanistic framework, but causal relationships remain to be established.

Furthermore, while we detected significant differences in plasma ARG1 protein levels between RR and RS pigs, the tissue origin of plasma ARG1 cannot be definitively determined from our data. ARG1 is expressed not only in the liver but also in erythrocytes, where it is abundant and may be released into plasma during normal red blood cell turnover. To minimize this confounder, all plasma samples were visually inspected for hemolysis, and no samples with visible hemolysis were included in the analysis. However, we cannot completely exclude erythrocyte-derived ARG1 contributions. The observed metabolomic changes—particularly the reduction in ornithine and glutamine—are consistent with reduced hepatic urea cycle flux, supporting a hepatic origin of the functional changes. Nevertheless, direct measurement of ARG1 expression and activity in liver tissue (e.g., Western blot, qRT-PCR, and enzymatic activity assays) would be required to definitively establish that the plasma ARG1 differences reflect changes in hepatic urea cycle activity.

While our data strongly associate ARG1-mediated nitrogen reprogramming with growth advantage, several limitations should be acknowledged. First, the multi-omics analyses were performed on a relatively small sample size (*n* = 6 per group), although this is common in deep plasma proteomic studies and the effect sizes were large. Second, the absence of purebred Laiwu (LL) pigs as a control precludes distinction between true heterosis and direct genetic contribution from the Laiwu breed. Third, the proposed model relies partly on inferred biochemical pathways; direct measurements of NOS enzymatic activity, tissue blood flow, or mTOR phosphorylation in skeletal muscle would further strengthen causality. Fourth, all experimental animals were castrated males, and whether the same mechanisms operate in intact males or females remains unknown.

## 5. Conclusions

In conclusion, this study demonstrates that the growth superiority of Ronglai binary hybrid pigs is closely associated with a reprogrammed nitrogen metabolic network initiated by the downregulation of arginase-1 (ARG1). Integrated proteomic and metabolomic analyses revealed that ARG1 downregulation attenuates urea cycle flux, leading to arginine accumulation and reduced glutamine levels, with arginine-containing dipeptides serving as a potential buffer pool. Importantly, we provide direct experimental evidence that the accumulated arginine activates the NOS pathway, as reflected by significantly elevated plasma NOx levels in RS pigs. NO, in turn, may promote growth through multiple mechanisms, including vasodilation, mTORC1 activation, satellite cell proliferation, myostatin suppression, and enhanced mitochondrial biogenesis. The proposed “throttle, activate, recycle” model explains how nitrogen resources may be systematically redirected from urea excretion toward growth-supporting anabolic pathways.

Our findings provide the first direct link between the growth advantage of a local pig cross and ARG1-mediated nitrogen metabolic remodeling. The identification of ARG1 and the NOS pathway as key nodes in this regulatory network offers actionable molecular targets for precision breeding strategies aimed at developing feed-efficient pig hybrids without compromising desirable indigenous traits such as meat quality and disease resistance. Future functional studies—including ARG1 knockdown in hepatocytes, isotopic ^15^N-tracing to quantify nitrogen flux, and tissue-specific assessment of mTOR activation and myostatin signaling—are required to establish causality and to translate these findings into practical breeding applications.

## Data Availability

The raw data supporting the conclusions of this article will be made available by the authors on request.

## Supplementary Materials

The following supporting information can be downloaded at: https://www.mdpi.com/article/10.3390/ani16152344/s1, Supplementary Figure S1: PCA analyses showing group separation; Supplementary Figure S2: Correlation diagram among samples.
