# Supplementary material for: Integrative Proteomic and Metabolomic Analyses Reveal Arginase-1-Mediated Nitrogen Metabolic Reprogramming Associated with Growth Advantage in Ronglai Binary Hybrid Pigs
Source: Animals (Basel). 2026 Aug 1;16(15):2344. doi: 10.3390/ani16152344 (PMC13463462; doi:10.3390/ani16152344)
Supplement: Supplementary file 1 [file animals-16-02344-s001.zip › animals-4410839-supplementary.pdf]

# Integrative Proteomic and Metabolomic Analyses Reveal Arginase-1-Mediated Nitrogen Metabolic Reprogramming Associated with Growth Advantage in Ronglai Binary Hybrid Pigs

**Wei Yang [1,2], Lan Wu [3], Hongyu Xie [3], Ding Zhang [3], Yuqin Wang [3], Jiajia Pan [3], Xinyi Xu [4], Xiaoqing Bai [1,2,*], Wei Zhang [3] and Yueting Zheng [3,*]**

1. Chongqing Academy of Animal Sciences, Rongchang, Chongqing 402460, China; yangw@cqaa.cn
2. Chongqing Seed Pig Farm Co., Ltd., Rongchang, Chongqing 402460, China
3. Key Laboratory of Animal Biochemistry and Nutrition, Ministry of Agriculture, Key Laboratory of Veterinary Biotechnology of Henan Province, College of Veterinary Medicine, Henan Agricultural University, Zhengzhou 450046, China; wl999633@stu.henau.edu.cn (L.W.); xiehongyu@stu.henau.edu.cn (H.X.); dingzhang@stu.henau.edu.cn (D.Z.); wangyuqin@stu.henau.edu.cn (Y.W.); first2017@henau.edu.cn (J.P.); zhangweiest@126.com (W.Z.)
4. Center for Biomedicalphotonics and Molecular Imaging, Xi'an Key Laboratory of Intelligent Sensing and Regulation of Trans-Scale Life Information, School of Life Science and Technology, Xidian University, Xi'an 710126, China; xyxu@xidian.edu.cn

* Correspondence: cqaav@163.com (X.B.); yuetingzheng@henau.edu.cn (Y.Z.)

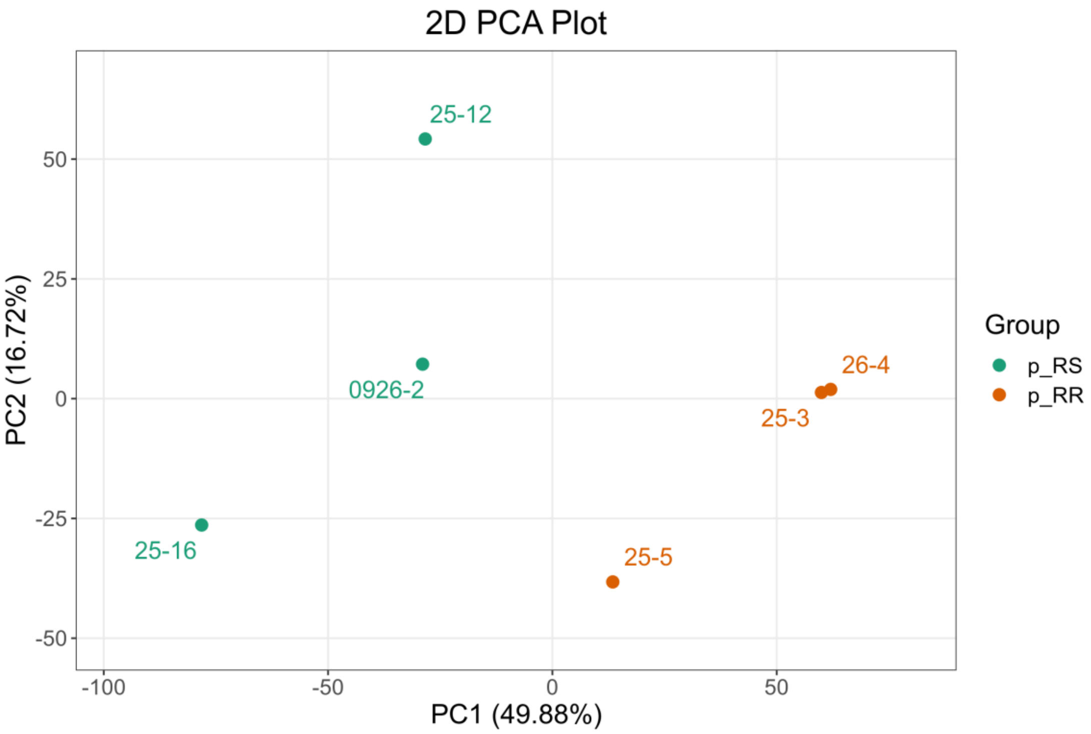


Figure S1 PCA analysis

Note: The horizontal and vertical axes represent the first and second principal components, respectively, and the percentages in parentheses indicate the contribution of each principal component to the sample variance; each point in the graph represents a sample, with different colors indicating different groups of samples.

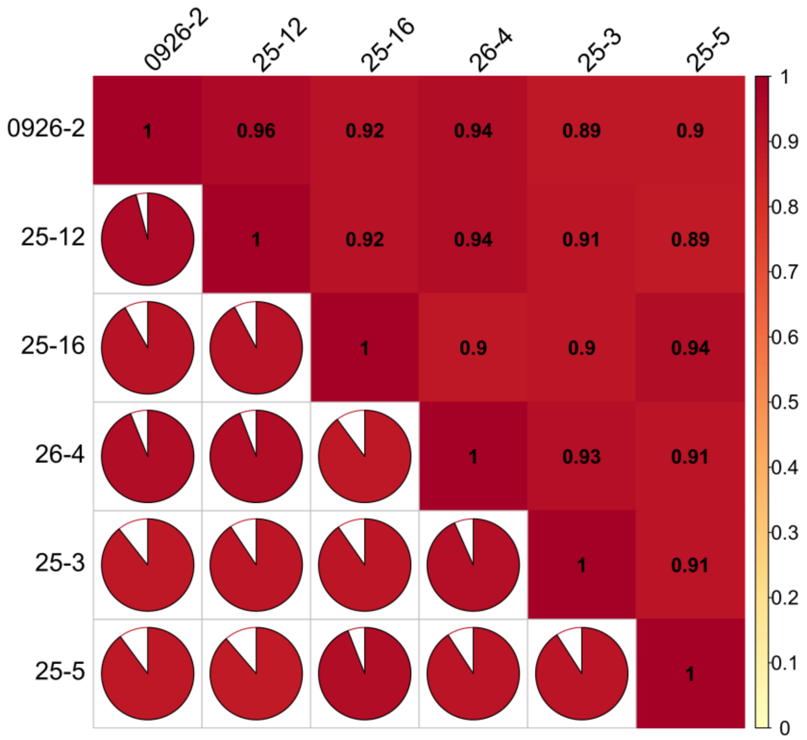


Figure S2 Correlation diagram among samples

The horizontal and vertical axes represent sample names, and the change from red to yellow in color indicates the change from high to low correlation. The size of the sector area in the figure represents the magnitude of the correlation coefficient between the corresponding horizontal and vertical axis samples; the numbers in the figure represent the corresponding correlation coefficients between the horizontal and vertical axis samples.
